# Feasibility and Merits of Performing Preclinical Imaging on Clinical Radiology and Nuclear Medicine Systems

**DOI:** 10.1155/2013/923823

**Published:** 2013-12-30

**Authors:** Mehmet Bilgen

**Affiliations:** Biophysics Department, Faculty of Medicine and Bioimaging Research Center, Erciyes University, 38039 Kayseri, Turkey

## Abstract

*Aim*. Researchers have limited access to systems dedicated to imaging small laboratory animals. This paper aims to investigate the feasibility and merits of performing preclinical imaging on clinical systems. *Materials and Methods*. Scans were performed on rat and mouse models of diseases or injuries on four radiology systems, tomosynthesis, computed tomography (CT), positron emission tomography/computed tomography (PET-CT), and Magnetic Resonance Imaging (MRI), based on the availability at the author's institute. *Results*. Tomosysthesis delineated soft tissue anatomy and hard tissue structure with superb contrast and spatial resolution at minimal scan time and effort. CT allowed high resolution volumetric visualization of bones. Molecular imaging with PET was useful for detecting cancerous tissue in mouse but at the expense of poor resolution. MRI depicted abnormal or intervened tissue at quality and resolution sufficient for experimental studies. The paper discussed limitations of the clinical systems in preclinical imaging as well as challenges regarding the need of additional gadgets, modifications, or upgrades required for longitudinally scanning animals under anesthesia while monitoring their vital signs. *Conclusion*. Clinical imaging technologies can potentially make cost-effective and efficient contributions to preclinical efforts in obtaining anatomical, structural, and functional information from the underlying tissue while minimally compromising the data quality in certain situations.

## 1. Introduction

Research institutes and pharmaceutical industry have been adopting *in vivo* preclinical imaging technologies to sustain their cutting edge bioscience research with translational focus and accelerate drug discovery processes [1–4]. Varieties of dedicated systems were manufactured by a number of vendors and are currently in use for imaging small laboratory animals (rodents: rat or mouse) at high quality, sensitivity, specificity, and resolution [5, 6]. In developing countries, however, access to such platforms has been limited to none because of a number of reasons including equipment costs or small number of ongoing research projects, not justifying their installations. The investigators of these countries were therefore put in a disadvantaged position compared to their counterparts in the developed countries. Installing a centralized small animal imaging facility within the country or a geographical region would have been an option to rectify the issue, but this has yet to be realized as in the case of the author's current country of employment. In those instances where the research or discovery demands preclinical imaging, but a special system for performing the task is lacking; the use of clinical radiology systems installed in a typical university hospital has been considered as a viable, but challenging, alternative to support the ongoing preclinical research [7–11]. This paper therefore aims to demonstrate the strategic value of such practice and its merits in the sense that the acquired images may not be the highest grade but with sufficient qualifications to meet the preclinical imaging needs. Particularly, the study involved four clinical systems (Siemens tomosynthesis, Toshiba computer tomography (CT), hybrid Philips positron emission tomography and CT (PET-CT), and Siemens magnetic resonance imaging (MRI)), all installed at the author's institute. Images acquired with each system were presented from the scans performed on rat and mouse models of diseases or injuries employed in different research projects. The key features of the systems were introduced and the scan protocols used for the acquisitions with specific sets of parameters were described. Practical aspects of making additional modifications and improvements (slight or minimal) on the systems and scan procedures were analyzed and discussed for gathering optimal anatomical, structural, functional, and molecular data. Methods were suggested for overcoming the limitations of the systems within the context of increasing scan performances in longitudinal studies involving multiple imaging modalities. Extreme modifications in hardware, such as gradient coil insertion or software upgrades, were excluded from the scope of the paper, but additional gadgets or considerations were discussed for longitudinally scanning small animals under anesthesia while monitoring their vital signs.

## 2. Materials and Methods

**Figure 1:**
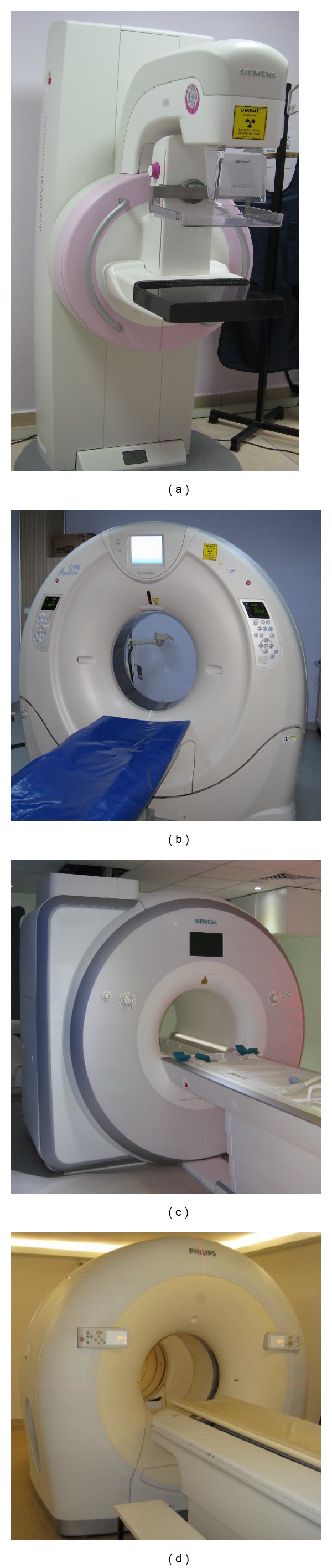
Clinical radiology systems: (a) tomosythesis, (b) computer tomography, (c) magnetic resonance imaging, and (d) positron emission tomography/computer tomography used in this study.

**Figure 2:**
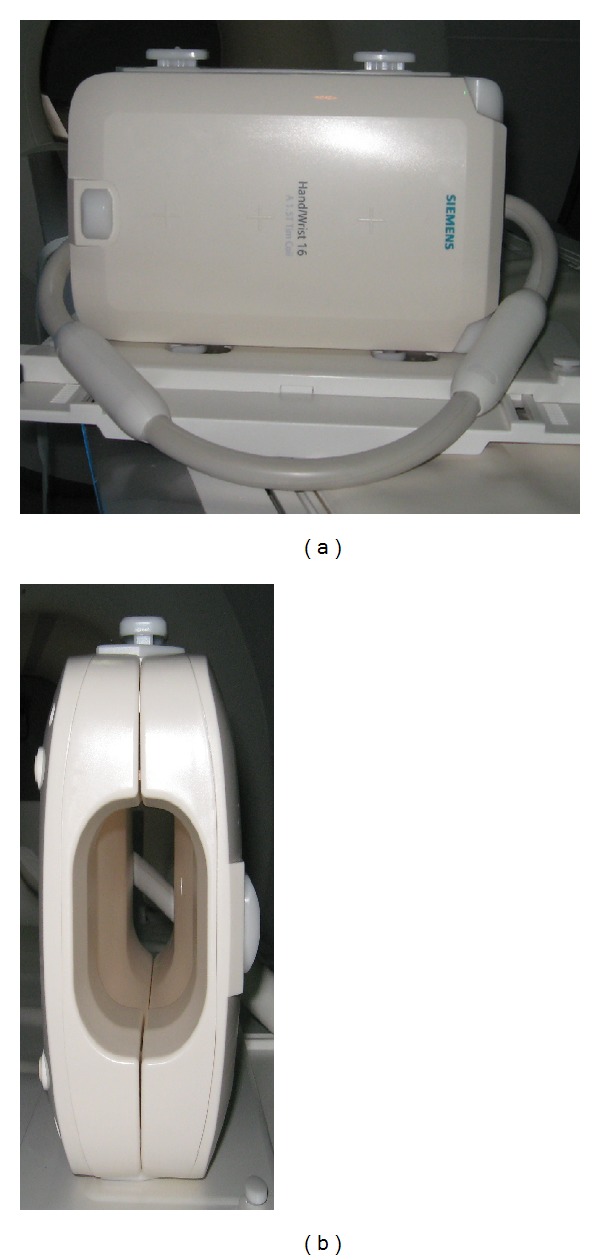
Human/wrist coil used for MRI of mouse and rat.

The scans were performed off hours using four clinical systems, shown in Figure 1, that were installed at the author's institute on postmortem rat or mouse models of disease or injury as parts of the ongoing research projects. One of the systems was a new digital mammography device tomosynthesis (Siemens) with capability to image calcified dense tissue of breast with clarity, using low dose X-ray radiation. The device creates images with minimal tissue overlap by acquiring data from limited angle rotation of the X-ray source. It was originally developed for improving the outcomes of screening with routine mammography. It potentially increases the cancer detection rate by decreasing false negatives and false positives. The second clinical system was a 320 slice CT scanner (Toshiba Aquilion One). In this system, the image slices were acquired with full rotation of the X-ray source and detector around the body. The third system was a combination of positron emission tomography and CT (Philips Gemini TF 16). The CT data were used as background which is overlaid by the PET data to form fusion image. The fourth system was a 1.5 T whole body MRI scanner (Siemens Magnetom Aera, A Tim and Dot System) with 70 cm bore diameter and 45 mT/m gradient coil. MRI data were acquired using a wrist coil (Hand/Wrist 16, A 1.5 T Tim coil) in Figure 2 to increase the signal-to-noise ratio (SNR). All systems were connected to a common picture archiving and communication (PACS) unit and data were available in DICOM format.

We tested the consistency, quality, and repeatability of the data acquisitions on all systems using multimodal scans performed multiple times from normal and pathologically abnormal animals. The acquisitions were repeated 5 times in each system after removing the normal animal and then placing it back into the system. Other tests were also carried out similarly in model animals. A mouse model of intraperitoneal tumor was repetitively scanned 5 times by following the imaging procedures from start to end using tomosynthesis system. CT images were acquired 5 times back to back from a rat with craniotomy. PET-CT imaging was also performed 5 times on a mouse with mammalian tumor. MRI involved similar 5 investigations on a rat whose different brain regions were injected with a cocktail of pharmacological and contrast agents. Sampled examples from these tests were provided in the next section.

## 3. Results

**Figure 3:**
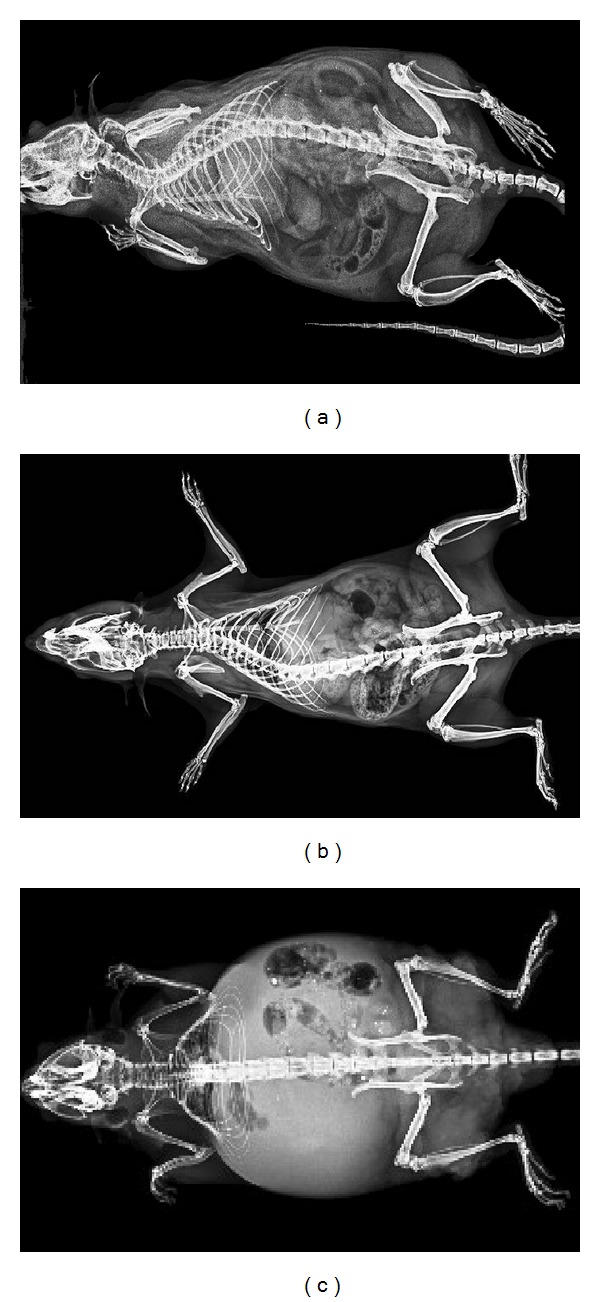
Images acquired in coronal view with tomosythesis system: (a) normal rat, (b) normal mouse, and (c) mouse with peritoneal tumor. kV = 32, mA = 78, number of images = 26, FOV = 305 mm × 239 mm, and isotropic pixel resolution = 0.085 mm.

Figure 3 shows images acquired from a normal rat, a normal mouse, and a mouse model of intraperitoneal tumor using tomosynthesis system. The images clearly depict the peritoneal soft tissue and bones with a superb contrast at high spatial resolution.

**Figure 4:**
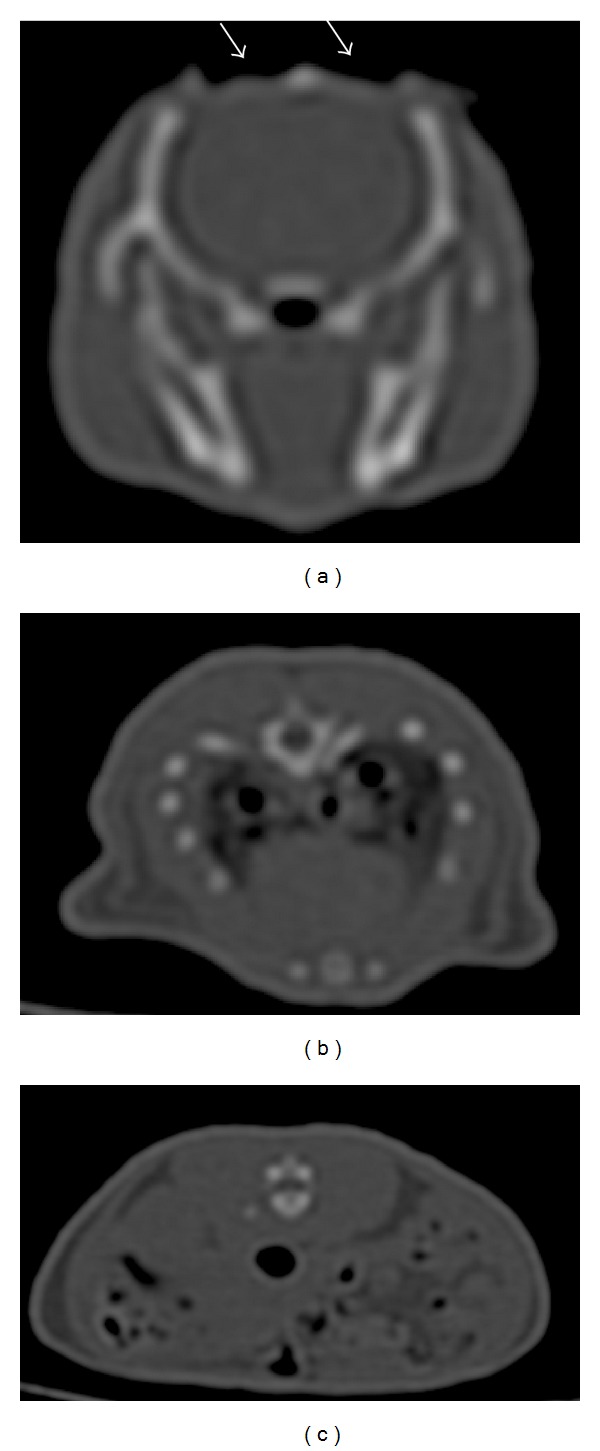
Axial images acquired from a rat with craniotomy using Toshiba Aquillion One CT scanner. Helical scan, kV = 120, mAs = 100, slice thickness = 0.5 mm, and image matrix = 512 × 512 pixels. Arrows point to bilateral craniotomy.

**Figure 5:**
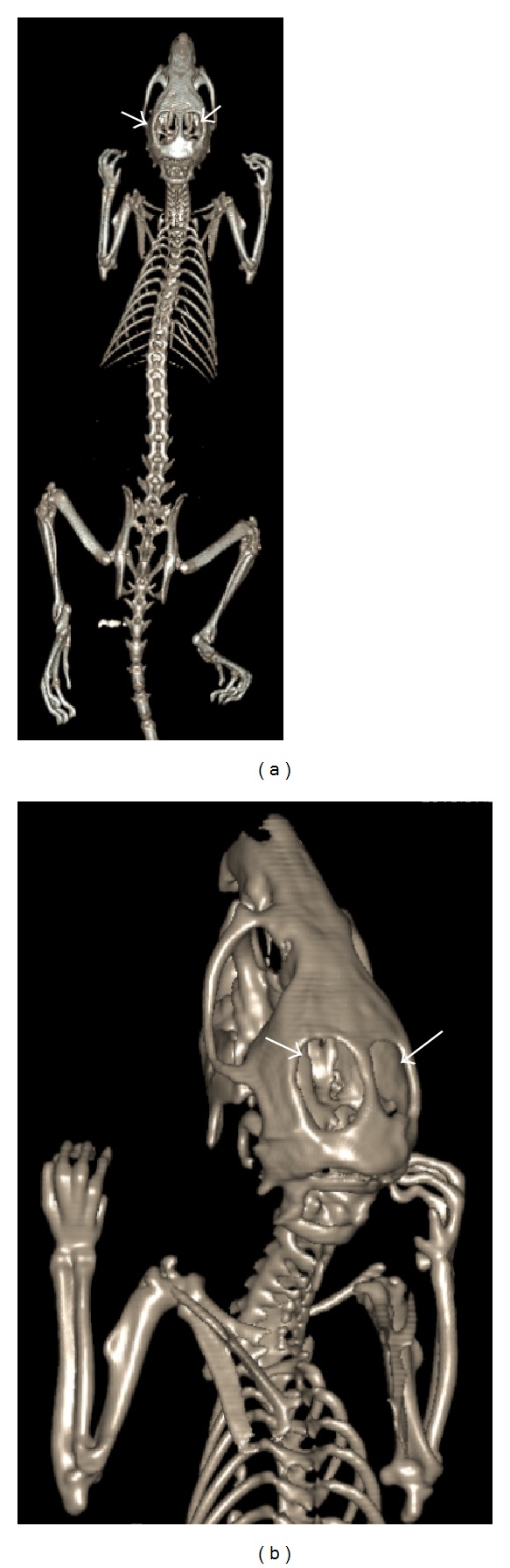
Rat skeleton constructed from the CT images in Figure 4 using vendor supplied software. Arrows point to bilateral craniotomy.

**Figure 6:**
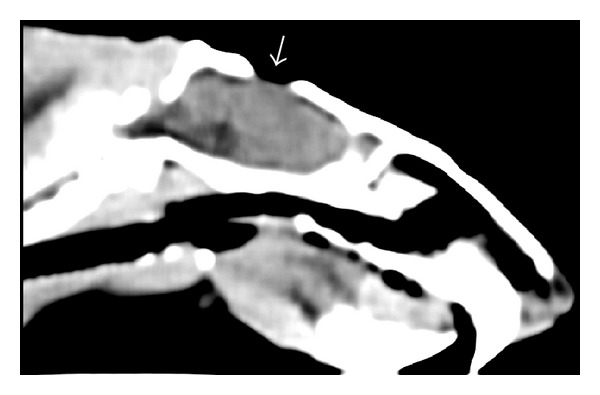
Visualization of 3D volumetric CT data sliced in sagittal plane using vendor supplied software. Arrow points to craniotomy.

Figures 4–6 display the CT images of a rat that has received craniotomy for intraparenchymal electrode recording and localized drug delivery to the brain. Hyperintensity in the images represents strong attenuation of X-ray, as a characteristic property of calcified tissue like the bone. The soft tissue contrast in the CT images, however, appears minimal due to the similarities between the X-ray attenuation properties of soft biological tissues. With the help of vendor supplied software, segmentation based on intensity threshold followed by postprocessing produced the volumetric view in Figure 5. The 3D visualization better delineated the bones of the skeleton and hence the extent of craniotomy in the skull. The software also enables reorganizing or slicing the 3D data in any selected oblique plane.  Figure 6 shows the rat brain in sagittal plane after such postprocessing.

**Figure 7:**
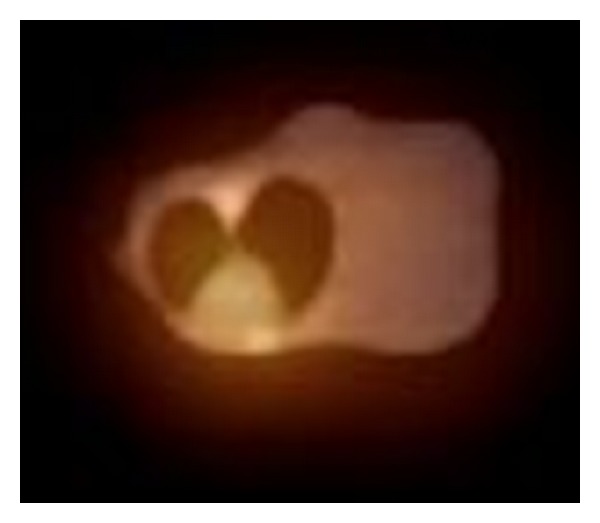
PET-CT fusion image of mouse with mammalian tumor. CT parameters: kV = 90, mAs = 20, slice thickness = 2 mm, FOV = 35 × 35 cm, and image matrix = 512 × 512 pixels. ^18^F was delivered IV in the amount of 19.28 MBq.

Figure 7 shows PET-CT fusion image of a mouse with mammalian tumor. The tone of red color represents the PET data overlaid on the CT background.

**Figure 8:**
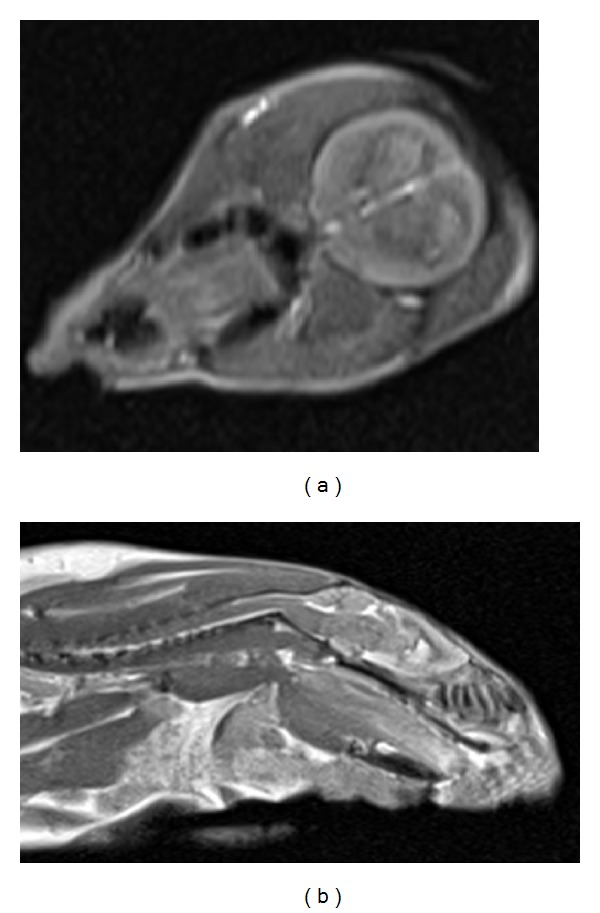
Axial and sagittal turbo spin echo images of rat head acquired with MRI scanner using the following parameters: TR/TE = 6440/14, slice thickness = 1.1. mm, FOV = 70 mm × 90 mm, image matrix = 140 × 256, turbo factor = 4, and NEXT = 1.

**Figure 9:**
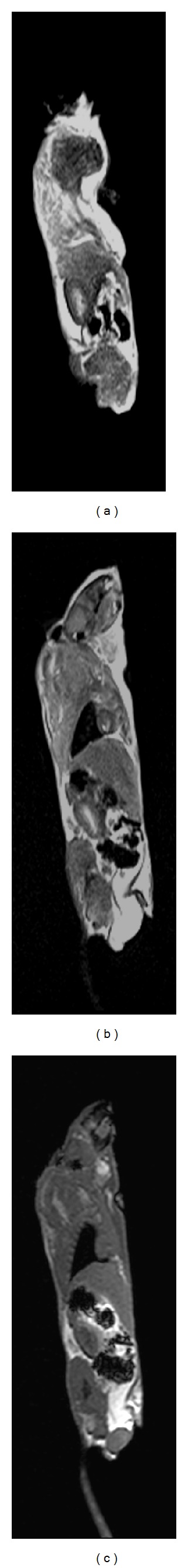
Sagittal turbo spin echo images of mouse with mammalian tumor. The acquisition parameters were TR/TE = 4000/56, slice thickness = 0.8 mm, FOV = 120 mm × 120 mm, image matrix = 224 × 320 pixels, turbo factor = 4, and NEXT = 1.

**Figure 10:**
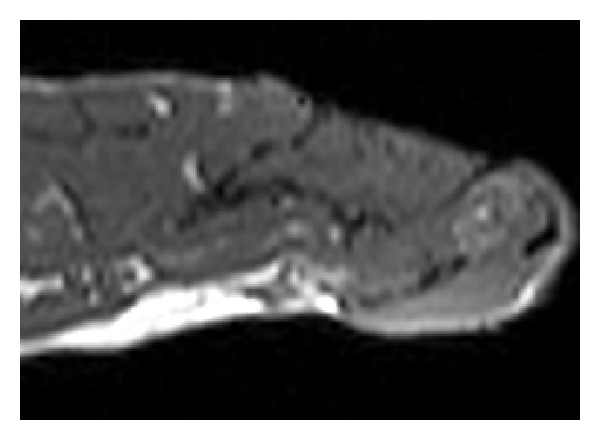
Sagittal T1-weighted gradient echo images of rat brain injected with pharmacological compound in conjunction with Gadolinium contrast agent using micropipette. The acquisition parameters were TR/TE = 15/4.76 ms, slice thickness = 0.8 mm, FOV = 109 mm × 218 mm, image matrix = 128 × 256 pixels, and NEXT = 1.

Figures 8–10 are the MRIs of a rat brain and a mouse body. The images in Figure 8 are from a normal rat, but the one in Figure 10 shows traces of micropipette used for local injection of a pharmacological agent in conjunction with MR contrast agent Gadolinium into different brain regions. Gadolinium was delivered in large concentration and hence produced locally hypointense signal (as opposed to hyperintensity, as seen in the figure) due to emphasizing T2 effect rather than T1 enhancement in the regions where it is present [12].

## 4. Discussion

Beyond clinical radiology, *in vivo* evaluation of biological tissue of interest is required in biomedical research for demonstrating a particular point of view or gathering additional information to ultimately leverage or strengthen research findings suitable for promoting research or publication of its outcome. One way to evaluate the underlying tissue has been based on the principle of “seeing is believing,” which led to the broad field of preclinical imaging. As it aims to advance fundamental concepts to create knowledge by bringing individuals from different backgrounds, preclinical imaging is considered truly multidisciplinary and integrated. In simple terms, imaging can be described as an art of producing contrast to convey information. Many modalities based on different physical principles and contrast mechanisms coupled with proper equipment have been developed along for visualizing soft tissue and subsequently for cross comparison and verification [3]. With each modality, the mechanism through which image contrast is generated varies but relies on either endogenous or exogenous processes. Endogenous contrast uses specific physical properties internal to the tissue, but exogenous contrast is produced by delivering specially formulated external agents into the tissue as in the case of targeted agents developed for improving the specificity (MICAD: molecular imaging and contrast agent database, http://www.ncbi.nlm.nih.gov/books/NBK5330/). Unfortunately, most of the past developments concerning the commercial equipment and supplementary tools for preclinical imaging have yet to find their way into many institutes to serve the needs of investigators therein. Such handicaps, however, did not discourage attempts of small animal imaging on the available diagnostic radiology or nuclear medicine equipment in the past [7–11]. As it was the objective of this investigation, performing multimodal preclinical imaging was demonstrated to be a viable option in clinical equipment but with varying degrees of merits as well as pertaining to certain challenges. The imaging hardware as well as the acquisition and postprocessing software in the systems were sophisticated enough to successfully perform the tasks by the skilled users of the systems.

Based on our experience and the acquired data, the new line of mammography scanner, tomosythesis, is particularly encouraging for preclinical imaging as this system could be instrumental in studies that require enhanced quality and resolution at minimal scan time. The contrast produced in output images was sufficient enough to distinguish soft tissue types in addition to bones (Figure 3). Viewing the subject was, however, restricted only to a limited number of planes. The complete visualization of subject in 3D was possible with CT technology with great specificity to the elements of skeleton (Figure 5) and MR with contrast sensitive to soft tissue (Figures 8 and 9). In CT, the subject can be viewed from a plane with arbitrary orientation but after digitally interpolating the 3D data set. On the other hand, direct visualization across any plane is possible with MRI. For MRI, it is advisable to use the smallest size RF coil, such as wrist coil or knee coil, to increase the SNR and hence, resolution of the images [13, 14]. Within all the scanners, PET images had the lowest range in terms of image resolution.

All scanners considered in this study were calibrated and geared towards diagnostic imaging of humans in clinical environment. Small animal imaging in some cases exceeds the capabilities of the system and requires implementations of specific adjustments or considerations [15]. For example, MRIs presented above were obtained using basic spin-echo sequence. More complicated sequences, for example, echo-planar-imaging, place a heavy demand on the existing hardware. Imaging rat or mouse with these sequences strains the scanner to its limits, especially when the field of view is set to a small area. In addition, artifacts, such as ghosting, appear in the resulting images. To address these issues and determine the limits of the hardware, performance evaluation and optimization procedures can initially be carried out on each scanner using test phantoms manufactured by placing a dead mouse or rat in formaldehyde solution within a sealed tube. These phantoms can also serve for quality control purposes [14].

Compliance with the institutional regulations can be another critical concern to be addressed. In our experience, one concern was the contamination or biosecurity issue due to imaging rodents in clinical systems. Properly addressing this requires spending some time to convince the authorities and the institutional human and animal ethics committees. Meetings can be held to describe the methods to be used for handling the animals and special care to be exercised for preventing contamination in the scanner room and the environment. Another common concern was how to keep the animals out of sight from the patients or public. This issue can easily be resolved by implementations such as transporting the animals and performing the scans during off hours, during which no patients are scheduled for scanning.

Imaging rodents on clinical systems also involves practical considerations. For example, tomosythesis system is typically operated in automation mode during routine mammography. The system places a pressure plate over the breast and automatically compresses it to a preset level. Next, the overall composition of the subject is evaluated by an initial intensity test. Exposure to X-ray during this test measures the attenuation characteristics of the subject. Based on the result, the values are automatically set for the excitation parameters (kV and mA) of the X-ray source. If the attenuation is found to be significant, the scan process is aborted. We experienced such situations when imaging rats where the bones caused large attenuation. Strategically, small animals can be best imaged on tomosythesis by operating the system in manual mode. This allows adjusting the kV and mA parameters of the exposure freely without the involvement of the pressure plate. However, it is also important to note that, operating this and the other systems, personnel safety should not be compromised while increasing the efficiency and power of the imaging protocols.

Successfully performing scans on the systems and improving the outcomes require meeting other conditions as well [16]. For example, multimodal imaging necessitates securing the animal to a holder [17]. Assembling cradles for holding a rat or mouse from plexiglass material is advisable for maintaining compatibility with the magnetic field of the MRI scanner. For securing the animal to cradle, mouth and ear pieces can be incorporated into the design. For keeping the animal warm during scans, the features of the design can be enhanced by etching tubes in the section where the animal would rest for circulating heated water with regulated temperature. In some cases, warm air delivered by pipes may be sufficient enough to fulfill the same task [18]. Air approach would be advantageous since it would eliminate the fold over artifacts in MR images, originated from water in the tubes.

MRI and PET scans take longer time than those of tomosynthesis and CT. Logistically, performing long scan with gas anesthesia, for example, isoflurane, is more practical compared to using injectable anesthetic agents [19]. A nose mask attached to a plastic tube can be used for delivering the gas anesthesia. The tube is connected to an anesthesia machine at the other end. Making the tube long enough allows placing the anesthesia machine far away, especially beyond the 5 gauss magnetic field line of the MRI scanner.

When the animal is subjected to a long time scan, its vital physiological signs need to be monitored. The clinical systems are equipped with units for recording body temperature, EKG, and respiration signals but from humans [20]. But, such capabilities of clinical systems may not have much use in small animal studies unless modified or interfaced properly to detect the signals from the animal. For example, heart rates in rat and mouse are about 4 to 8 times faster than that of humans, and these high rates cannot be detected by the monitoring units of the clinical scanners. For these cases, specific hardware can be constructed for interfacing the animal's vital signals to the existing units. This would allow respiratory and cardiac gated image acquisitions. More sophisticated monitoring devices, some of which are compatible with MRI scanners, are also available commercially (http://www.m2mimaging.com, http://www.i4sa.com). However, connecting home-made interface gadgets or commercial monitoring devices to the scanner requires permission from the scanner manufacturer, which may involve reach agreements.

Another common issue when imaging across different platforms is the image registration issue. The use of cradle partially solves this problem, but further improvement in fusing multimodal images can be achieved by placing fictitious markers doped with PET, CT, and MR contrast materials on the animal and holder.

In conclusion, clinical radiology technologies can be considered as viable options for *in vivo* multimodal imaging of rodent models of human diseases or injuries. Critical information gathered from image-based qualitative or quantitative analysis can potentially make cost-effective and efficient contributions in preclinical research with translational focus. Successful operations would require special implementations of support facilities—including animal preparation and handling, specific holders, monitoring device, anesthesia, dosing apparatus, and logistical and procedural considerations for preventing animal-to-animal contamination and for decontamination and off-hour operations of the imaging equipment.
